# A displaced bronchus branching from the left main bronchus and bifurcating into the apicoposterior and anterior bronchi of the left upper lobe in a healthy adult male: A case report^☆^

**DOI:** 10.1016/j.radcr.2022.06.072

**Published:** 2022-07-27

**Authors:** Akira Katayama, Jiro Sato, Yosuke Kano, Yoko Mise, Satoshi Suzuki, Osamu Abe

**Affiliations:** a Department of Radiology, Graduate School of Medicine, The University of Tokyo, 7-3-1 Hongo, Bunkyo-ku, Tokyo, 113-8655, Japan; b Department of Radiology, Tokyo Metropolitan Police Hospital, 4-22-1 Nakano, Nakano-ku, Tokyo, 164-8541, Japan

**Keywords:** Left upper lobe, Displaced bronchus, Eparterial bronchus, Pulmonary artery, Accessory fissure, Anomaly

## Abstract

A displaced left upper bronchus is a rare anomaly. We report the case of a 45-year-old man with a displaced bronchus, branching from the left main bronchus and bifurcating into the apicoposterior and anterior segment bronchi of the left upper lobe. The displaced bronchus passed behind the left pulmonary artery. To our knowledge, 12 similar cases of displaced bronchi have been reported to date. Displaced bronchi are difficult to detect prospectively on computed tomography. However, evaluating the accessory fissures may help establish an accurate diagnosis.

## Introduction

Several anatomical variations of the bronchi and pulmonary arteries have been reported. According to a previous study, tracheobronchial anomalies occurred in approximately 0.64% of cases [1]. Based on previous computed tomography studies, the frequency of an anomaly of the left upper lobe bronchus ranged from 0.015% to 0.08% of cases [1], [2], [3]. In most of these studies, a displaced bronchus, directed toward the left upper lobe, ventilated the apicoposterior segment. This study features a displaced bronchus that bifurcated into the apicoposterior and anterior bronchi.

## Case report

**Fig. 1:**
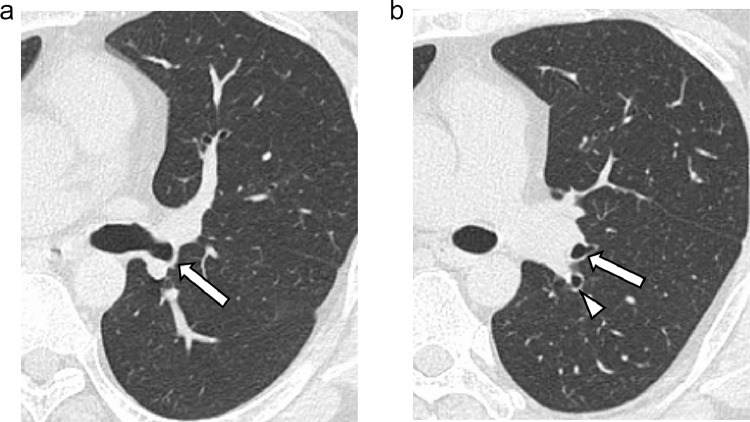
(A) A displaced bronchus (arrow) branches posteriorly from the left main bronchus behind the left pulmonary artery. (B) The displaced bronchus branches into the apicoposterior (arrowhead) and anterior (arrow) bronchi of the left upper lobe.

**Fig. 2:**
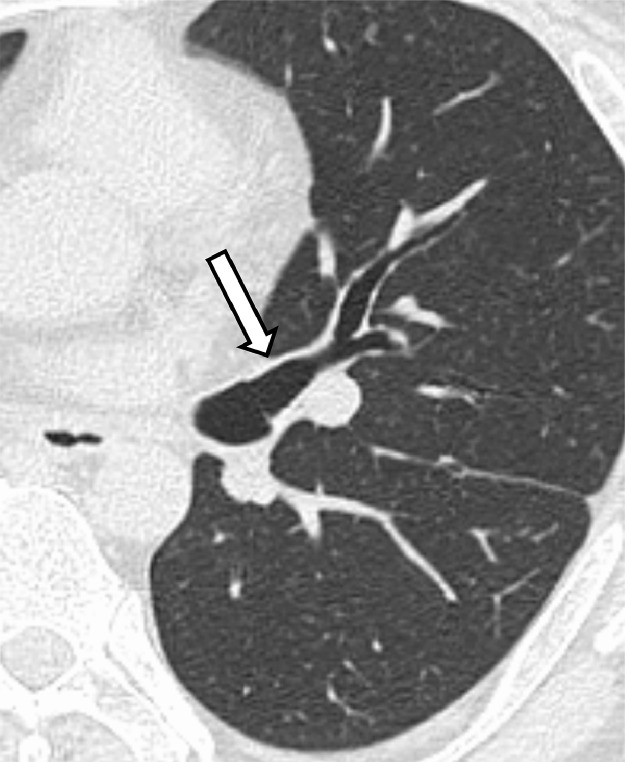
The lingual bronchus (arrow) branches anteriorly from the left main bronchus.

**Fig. 3:**
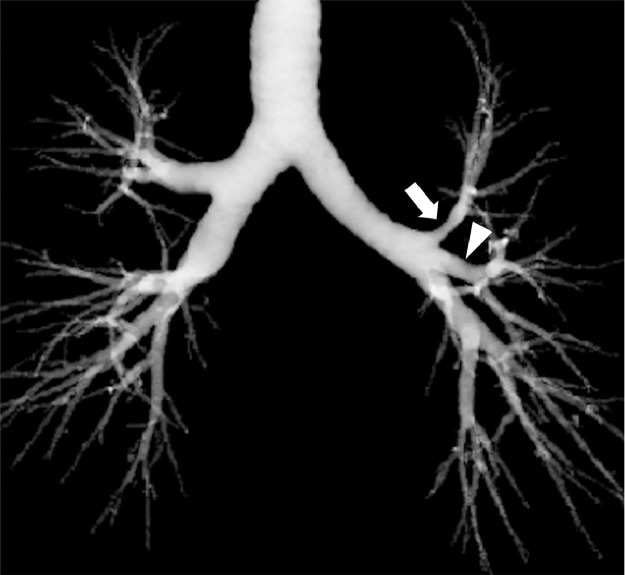
The displaced (arrow) and lingual bronchi (arrowhead) branch independently from the left main bronchus on a reconstructed computed tomography image of the tracheobronchial tree.

**Fig. 4:**
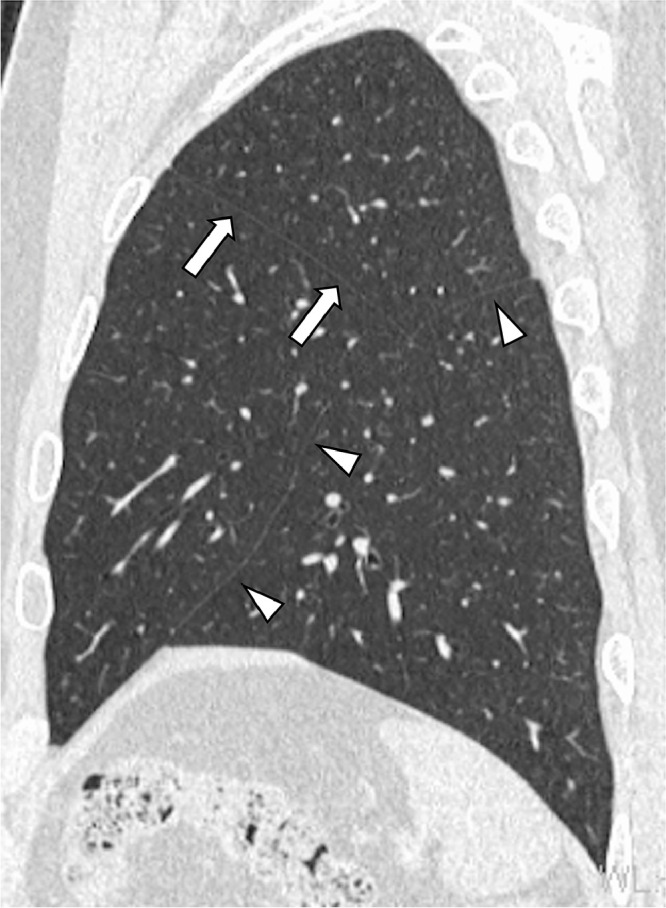
An accessory fissure (arrow) divides the left upper lobe into the lingula and the rest of the lobe on the reconstructed sagittal computed tomography image. A normal oblique fissure (arrowheads) is also present and the left lung is divided into 3 lobes.

A 45-year-old man with type 2 diabetes and hypertension was referred to our hospital with a suggestive abnormal shadow in the right lung field. He had no particular respiratory symptoms. The chest computed tomography examination findings showed no abnormalities in both lungs except for the displaced bronchus, branching from the left main bronchus behind the left pulmonary artery and bifurcating into the apicoposterior and anterior bronchi of the left upper lobe (Fig. 1). The left lingual bronchus independently branched from a more distal portion of the left main bronchus (Figs. 2 and 3). An accessory fissure divided the left upper lobe into the lingula and the rest of the lobe (Fig. 4). Anatomical variants were not observed in the right lung. Neither retroversion of the internal organs nor spleen abnormalities were observed.

## Discussion

According to Boyden, the bronchial branching anomalies of the left upper lobe are classified as a left eparterial bronchus or left prehyparterial bronchus [4]. A left eparterial bronchus refers to bronchi arising from the left main bronchus above the level where the left pulmonary artery crosses the left main bronchus. The left prehyparterial bronchus refers to bronchi originating from the left main bronchus between the level where the left pulmonary artery crosses the left main bronchus and the level of the left upper lobe bronchus. In the present case, the patient had a left eparterial bronchus.

The left eparterial bronchus is the most common left-sided bronchial branching anomaly. However, its incidence is 5 times less than that of the right preeparterial bronchus [5]. The prevalence of the left eparterial bronchus is approximately 1% based on anatomical studies and 0.3%-0.5% based on a bronchographic study [6]. The left eparterial bronchus usually ventilates the apicoposterior segment [5]. The patient in the present case had a rarer anomaly since the bronchus ventilated the apicoposterior and anterior segments. To our knowledge, there have been 12 previously reported adult cases with similar characteristics [6], [7], [8], [9], [10], [11], [12], [13], [14]. These patients presented the same type of displaced bronchus. Of these 13 patients (including our patient), 10 were male, and their ages ranged between 28 and 83 (median, 59) years. A displaced bronchus ran behind the left main pulmonary artery in all patients. The left lung was divided into 3 lobes in 9 patients, including the patient in the present case. Among these 9 patients, 6, including the present patient, had oblique and horizontal fissures. Four patients had 2 lobes, and 2 of these patients had incomplete accessory fissures. Twelve out of the 13 patients had a complete or incomplete horizontal fissure.

Accessory fissures in the left upper lobe were reportedly observed in 8%-18% of anatomical studies [4,15]. In a previous study, accessory fissures were detected in 7 out of 10 cases of the left eparterial bronchus. The presence of an accessory fissure has been associated with abnormal branching of the bronchi and blood vessels [6]. The small number of reported cases of the left eparterial bronchus was likely attributed to the high number of missed cases. It is difficult to detect a left eparterial bronchus using a preoperative computed tomography scan. There have been studies, reporting the incidental intraoperative resection of the left eparterial bronchus [6,10]. As the left eparterial bronchus is associated with the presence of an accessory fissure, it may be used to increase the diagnostic accuracy for this anomaly. A radiologist's report will allow operators to perform procedures, such as bronchoscopy and surgery more cautiously.

The main differential diagnosis for a left eparterial bronchus is right thoracic isomerism [5,6], which is characterized by bilateral trilobar lungs, each with an eparterial bronchus [6]. The left and right main bronchi are of equivalent length [6]. Moreover, right isomerism is associated with bilateral right morphologic atria, asplenia, midline liver, and a variably located stomach [5]. Congenital heart diseases are common among patients with right isomerism. It has been associated with mortality rates of 80%-90% during the first year of life [5]. In the present case, the patient's left and right main bronchi had different lengths, and there were no noted abdominal abnormalities.

This study reported the case of a displaced bronchus, bifurcating into the apicoposterior and anterior bronchi of the left upper lobe. It is important to detect this bronchial branching anomaly to facilitate safer invasive procedures, such as bronchoscopy or lobectomy.

## Patient consent

Informed consent for patient information to be published in this article was obtained.
